# Incidence and predictors of left ventricular remodeling among elderly Asian women: a community-based cohort study

**DOI:** 10.1186/s12877-017-0411-x

**Published:** 2017-01-14

**Authors:** Jing Wu, Caiqin Wu, Wenjing Fan, Jie Zhou, Ling Xu

**Affiliations:** 1 School of Nursing, Shanghai University of Traditional Chinese Medicine, 1200 Cai Lun Road, Shanghai, People’s Republic of China; 2 Yueyang Hospital of Integrated Traditional Chinese and Western Medicine, Shanghai University of Traditional Chinese Medicine, 110 Ganhe Road, Shanghai, People’s Republic of China

**Keywords:** Left ventricular enlargement, Left ventricular hypertrophy, Predictor, Cohort, Women, Elderly

## Abstract

**Background:**

Left ventricular (LV) remodeling is closely linked to the progression of heart failure. There are limited data on the epidemiology of new onset LV remodeling among elderly women, which requires further investigation.

**Method:**

We examined data from a community-based cohort of women aged > 65 years, who had received > 2 echocardiography scans from 2009 to 2014. Exclusion criteria for patients included prior echocardiographic evidence of left ventricular enlargement (LVE) or hypertrophy (LVH). LVE was defined as the index of left ventricular internal diameter at end-diastole to height, and LVH was defined as the left ventricular mass and thickness index which indicate hypertrophy.

**Results:**

Of the 474 subjects (age 71.85 ± 6.47 years), 49 (10.3%) developed LVH, while 55 (11.6%) developed LVE during the mean follow-up period of 5 years. Independent predictors of LVH included: central blood pressure (CBP, per 10 mmHg) [HR 1.094, 95% CI 1.011–1.202], BMI˃25(kg/m ^2^)[HR 1.306, 95% CI 1.175–1.434], B-type natriuretic peptide (BNP) ≥ 100 (pg/mL) [HR 1.635, 95% CI 1.107–3.311] and brachial-ankle pulse wave velocity (baPWV) ≥16 m/s [HR 1.605, 95% CI 1.474–2.039]. Predictors of LVE were CBP (per 10 mmHg) [HR 1.121, 95% CI 1.027–1.238], BMI˃25(kg/m ^2^)[HR 1.302, 95% CI 1.173–1.444], Low-density lipoprotein cholesterol (LDL-C) [HR 1.193, 95%CI 1.013–1.405] and E/e’ ratio [HR 1.077, 95% CI 1.017–1.140].

**Conclusion:**

CBP and BMI were demonstrated to be independent and robust predictors of left ventricular remodeling among elderly women, including both LVE and LVH. BNP and baPWV were specifically related to the development of LVH, whereas LDL-C and E/e’ ratio were related to LVE.

## Background

Left ventricular (LV) remodeling is considered as a precursor for worsening cardiac function and is often associated with a high mortality rate in affected patients [1]. Studies have suggested that patients usually progress through an adverse LV remodeling phase prior to development of symptomatic heart failure (HF) [2]. Substantial progress has been made in improving the accuracy and early assessment of LV remodeling. However, identification of the early predictors of LV remodeling as well as their potential roles is still poorly understood. Recently, several longitudinal cohort studies have identified the importance of chronic non-cardiac diseases which may result in the progression and deterioration of subclinical LV remodeling to HF [3, 4].

Numerous studies support the notion that males are significantly more at risk in the development of cardiovascular events. A potential underlying mechanism may be closely related to the protective effect of estrogen expression in women [5]. In the elderly population, the prevalence of HF is indistinguishable between males and females [6]. It is noteworthy that women are more prone to have increased relative wall thickness [7]. However, epidemiological data on the prevalence of LV remodeling risk factors and their impact in women are still unclear. Therefore, it is important to acquire data from population-based studies on women with LV remodeling, including incidences of LVE and LVH.

There are limited studies which examine the relationship between various bio-markers and central hemodynamic indexes with the specific type of LV remodeling. Our previous study demonstrated that women have double the incidence rate of LV remodeling compared to men [8]. Therefore, the current cohort study consisted of 474 elderly women subjects. This study aimed to explore potential predictors of new onset LV remodeling in women.

## Methods

### Study population

**Fig. 1:**
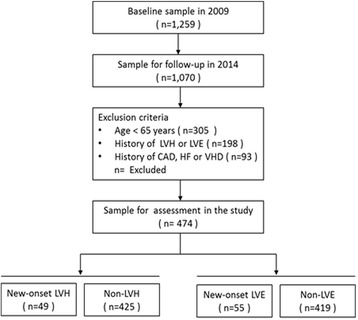
Flowchart of the study. LVH, left ventricular hypertrophy; LVE, left ventricular enlargement; CAD, coronary artery disease; HF, heart failure; VHD, valvular heart disease

The baseline examination was carried out in 2009. All the participants underwent physical examination, structured interview, blood sampling, and pulse wave analysis at the research center, as well as an echocardiography scan [8]. A total 1259 women (age>18) were randomly recruited from the Lujiazui community, Shanghai, China. 1070 of them underwent follow-up investigation in 2014. For the present study, we aimed to investigate the predictors of new-onset LV remodeling among elderly women. According to the exclusion criteria: 305 were excluded for their age less than 65 year; 93 were excluded for their medical history of Coronary heart disease (CHD), Heart failure (HF) and Valvular heart disease (VHD). The 198 subjects who were diagnosed as LV remodeling prior to cohort entry were also excluded. As a result, 474 subjects were eligible for the current study. During the 5 year follow up period, 45 women had developed LVH and 55 had developed LVE. Detailed information of the study population is provided in Fig. 1.

### Echocardiographic analysis

Echocardiography examination with trans-thoracic two-dimensional (2D) scanning was performed using a color Doppler ultrasonic device equipped with a 1.0–5.0 MHz transducer (GE Vivid 7; General Motors Corporation, New York, USA). Cardiac parameters including left atrium diameter (LAD), left ventricular internal diameter at end-diastole (LVIDd), left ventricular ejection fraction (LVEF), inter ventricular sepal thickness (IVST) and posterior left ventricular wall thickness (PVWT) were collected based on echocardiographic examination. E/A ratio was calculated based on peak early diastolic flow velocity (E velocity)/peak velocity of atrial contraction (A velocity). E/e’ ratio was determined by the E velocity/velocity of mitral annulus early diastolic motion (e’).

### Defining left ventricular enlargement and hypertrophy

We have previously reported the methodology for defining LVH and LVE in a cross-sectional study [8]. Thus, we used these former standards for the follow-up assessment. Preliminary criteria for defining LVH depended on the measurement of left ventricular mass index (LVMi). LVMi was determined by dividing the LVM measurement by height^2.71^. LVM was calculated based on the guidelines from the American Society of Echocardiography: LVM (g) = 0.8 × {1.04 × [(LVIDd + PVWT + IVST) ^3^− (LVIDd) 3]} + 0.6 (g) [9]. LVH was defined as LVMi > 46.7g/m^2.71^ for women and LVE was determined by indexing LVIDd to height, as previously demonstrated [10]. LVE was defined by LVIDd (mm) of greater than 28.3 + [13.9 × height (m)] for women [10].

### Laboratory measurements

All blood samples were obtained during the morning fasting state. Plasma total cholesterol (TC), high-density lipoprotein cholesterol (HDL-C), triglycerides (TG) and Fasting plasma glucose (FPG) were measured enzymatically. Brain natriuretic peptide (BNP, ECLIA Roche Diagnostics, Mannheim, Germany) was measured using electro chemiluminescence immunoassay. Glaciated hemoglobin (HbA1C) was measured using ion-exchange high performance liquid chromatography. Serum high-sensitivity C reactive protein (hsCRP) was measured using immunonephelometry (Dade Behring Diagnostics, Marburg, Germany). Homocysteine (HCY) was measured using fluorescence polarization immunoassay (Abbott IMx System, Chicago, IL, USA). Diabetes was defined as fasting plasma glucose (FPG) levels ≥7.0mmol/L, or self-reported diagnosis of diabetes. Dyslipidemia was defined as total cholesterol (TC) >5.7 mmol/L or low-density lipoprotein cholesterol (LDL-C) >3.6 mmol/L.

### Pulse wave analysis

Non-invasive pulse wave analysis was performed with an automated oscillometric device (HEM-9000 AI; Omron Corp., Tokyo, Japan). Subjects were examined in a sitting posture. Radial augmentation index (AI), Systolic blood pressure(SBP), diastolic blood pressure (DBP), and estimate of central blood pressure (CBP) were obtained after resting for 5–10 min. Hypertension was defined as either systolic blood pressure (SBP) ≥140 mmHg, diastolic blood pressure (DBP) ≥90 mmHg, or usage of antihypertensive medication. The detailed methodology was validated in our previous report [8].

### Brachial-ankle pulse wave velocity measurement

baPWV was measured using a volume-plethysmographic device (PWV/ABI, Omron Corp., Tokyo, Japan), which simultaneously recorded brachial and posterior tibial arterial wave forms with automated oscillometric method [11]. Echocardiographic examination was performed by placement of electrodes on the wrists and cuffs on the bilateral brachia and ankles of subjects, while resting in a supine position. The average value of baPWV was taken if there were differences in the measurements from the left and right sides [12].

### Statistical analysis

SPSS 19.0 (SPSS, Inc., Chicago, IL, USA) was used for statistical analysis. Categorical variables were presented as frequency percentages and the comparison between groups were based on the chi-squared test. Continuous variables were presented as means and standard deviation (SD) for normal distribution. Two samples T-test was used to compare differences between groups. We described skewed distribution variables as median (inter-quartile range). Accordingly, these variables were compared by nonparametric Mann-Whitney U-test. Multivariate cox proportional hazards regression models were developed to assess independent predictive value of LVE or LVH. A value of *p*<0.05 was considered statistically significant.

## Results

The baseline characteristics of the study population and patients with LVH or LVE are summarized in Tables 1 and 2. The average age of the 474 participants were 71.85±6.47 years, and had no prior history of heart diseases and echocardiographic evidence of LVH and LVE. Over a mean follow-up period of 5 years, 49 (10.3%) subjects had developed LVH; 55 (11.6%) subjects had developed LVE; 22 (4.6%) subjects had both LVH and LVE.

**Table 1:** Baseline clinical characteristics of study population according to left ventricular remodeling status at follow up (left ventricular hypertrophy vs. non-left ventricular hypertrophy, left ventricular enlargement vs. non-left ventricular enlargement)

Characteristic	All subjects (*n* = 474)	Groups		*P*-value	Groups		*P*-value
		LVH (*n* = 49)	Non-LVH (*n* = 425)		LVE (*n* = 55)	Non-LVE (*n* = 419)	
Age (years)	71.85±6.47	72.39±6.54	71.80±6.49	0.148	72.88±6.27	71.74±6.48	0.046
BMI (kg/m^2^)	24.35±3.18	27.08±3.47	24.03±2.98	0.000	26.95±3.72	24.00±2.93	0.000
SBP (mmHg)	138.85±20.50	151.02±19.61	137.39±20.12	0.000	150.25±20.80	137.37±20.01	0.000
DBP (mmHg)	76.49±11.20	81.67±9.15	75.79±11.14	0.000	81.82±10.35	75.80±11.44	0.000
CBP (mmHg)	144.35±22.30	157.13±21.28	142.93±19.80	0.000	157.05±24.34	142.70±21.51	0.000
baPWV (cm/s)	16.32 (14.42, 18.67)	17.47 (14.93, 20.53)	15.21 (13.98, 18.44)	0.000	17.10 (15.13, 19.73)	16.21 (14.31, 17.98)	0.028
Hypertension	205 (43.2%)	29 (59.2%)	176 (41.4%)	0.022	31 (56.4%)	174 (41.5%)	0.058
Diabetes	48 (10.1%)	6 (12.2%)	42 (9.9%)	0.514	7 (12.7%)	41 (9.8%)	0.281
Dyslipidemia	121 (25.2%)	13 (26.5%)	108 (25.4%)	0.865	18 (32.7%)	103 (24.6%)	0.237
Laboratory tests							
TC (mmol/l)	5.31±0.96	5.37±1.06	5.30±0.95	0.820	5.30±1.07	5.31±0.95	0.964
TG (mmol/l)	1.77±0.39	1.82±0.23	1.77±0.41	0.750	1.87±0.64	1.76±0.58	0.479
HDL-C (mmol/l)	1.32±0.32	1.31±0.35	1.32±0.31	0.802	1.27±0.29	1.32±0.32	0.212
LDL-C (mmol/l)	3.28±0.75	3.24±0.63	3.28±0.56	0.758	3.72±0.70	3.22±0.83	0.015
FPG (mmol/l)	5.39±1.01	5.59±0.78	5.37±0.96	0.249	5.53±0.66	5.37±0.41	0.337
HbA1C (%)	5.98±0.98	5.92±0.56	5.99±1.01	0.553	6.08±0.54	5.88±0.79	0.169
BNP (pg/ml)	60.0 (35.0, 95.0)	75.0 (40.0, 133.0)	60.0 (35.0, 91.5)	0.016	70.0 (42.0, 123.0)	60.0 (35.0, 93.5)	0.254
HCY (umol/l)	11.0 (9.5, 14.0)	12.0 (10.0,14.0)	11.0 (9.0, 14.0)	0.354	11.0 (9.0, 14.0)	11.0 (9.0, 13.5)	0.750
hsCRP (mg/l)	1.35 (0.69, 2.59)	1.98 (0.99, 3.27)	1.31 (0.68, 2.51)	0.019	1.97 (0.99, 3.86)	1.30 (0.67, 2.34)	0.004
UA (umol/l)	310.0 (263.5, 361.5)	323.0 (286.0, 346.0)	309.0 (263.0, 363.0)	0.207	308.0 (268.0, 365.0)	310.0 (264.0, 361.0)	0.745

Continuous variables with normal distribution were expressed as the mean±SD. In case of skewed distribution continuous variables (containing baPWV, BNP, HCY, hsCRP, UA) were presented as median (inter-quartile range)

*BMI* body mass index, *BNP* B-type natriuretic peptide, *DBP* diastolic blood pressure, *FPG* fasting plasma glucose, *LVH* left ventricular hypertrophy, *LVE* left ventricular enlargement, *HCY* homocysteine, *HDL-C* high-density lipoprotein cholesterol, *hsCRP* high-sensitivity C-reactive protein, *LDL-C* low-density lipoprotein cholesterol, *SBP* systolic blood pressure, *TC* total cholesterol, *TG* total triglycerides

**Table 2:** Baseline echocardiographic characteristics of study population according to left ventricular remodeling status at follow up (left ventricular hypertrophy vs. non-left ventricular hypertrophy, left ventricular enlargement vs. non-left ventricular enlargement)

Characteristic	All subjects (*n* = 474)	Groups		*P*-value	Groups		*P*-value
		LVH (*n* = 49)	Non-LVH (*n* = 425)		LVE (*n* = 55)	Non-LVE (*n* = 419)	
LVMI (g/m^2.71^)	35.91 (30.41, 41.36)	51.42 (48.42, 56.53)	34.79 (30.09, 39.51)	0.000	47.21 (40.10, 55.12)	34.87 (30.0, 39.67)	0.000
LVM (g)	12	176.19±21.29	122.81±25.18	0.000	160.31±17.26	124.10±23.77	0.000
RWT	0.35±0.05	0.36±0.05	0.34±0.04	0.133	0.30±0.04	0.35±0.05	0.000
LVEF (%)	64.44±3.51	62.83±3.01	63.51±3.57	0.135	63.78±3.36	64.53±3.51	0.124
E/e’ ratio	12.5 (9.09, 16.6)	14.28 (11.11, 18.0)	12.5 (9.09, 16.66)	0.080	13.46 (11.5, 16.6)	12.5 (8.33, 16.6)	0.019
E/A ratio	1.12±0.32	1.02±0.18	1.12±0.24	0.088	0.96±0.22	1.14±0.16	0.127
LAD (mm)	34.73±5.41	36.31±3.81	34.50±5.51	0.006	36.71±4.56	34.46±5.64	0.003
LVIDd (mm)	46.66±4.13	50.73±3.79	46.17±3.89	0.000	52.62±2.70	45.86±3.61	0.000
PVWT (mm)	8.05±1.06	8.98±1.30	7.03±0.98	0.000	8.05±1.27	8.01±1.02	0.670
IVST (mm)	8.56±1.24	10.08±1.26	8.38±1.11	0.000	8.97±1.57	8.11±1.17	0.036

Continuous variables with normal distribution were expressed as the mean±SD. In case of skewed distribution continuous variables (containing LVMI and E/e’) were presented as median (inter-quartile range)

*LVH* left ventricular hypertrophy, *LVE* left ventricular enlargement, *LAD* left atrium diameter, *LVEF* left ventricular ejection fraction, *LVMI* left ventricle mass index, *LVIDd* left atrium diameter, left ventricular internal diameter at end-diastole, *IVST* inter ventricular sepal thickness, *PVWT* posterior left ventricular wall thickness

Comparison between patients with or without LVH at baseline indicated that the level of BMI, CBP, baPWV and hsCRP in patients with LVH was significantly higher than that in those without LVH (*P* <0.05 for all). A similar result was observed in patients with or without LVE. Of note, increased BNP level was only found in subjects with LVH. Patients with LVE had higher age and were prone to have increased levels of LDL-C. Cardiac ultrasonography parameters between these groups are displayed in Table 2. Owing to the echocardiographic definitions for LVE and LVH, both groups had higher left ventricular geometry parameters. Subjects with LVE were also observed to have higher E/e’ ratio.

The multivariate models were developed to predict the first events of LVH and LVE. We selected variables that demonstrated significant statistical differences between groups (LVH vs. non-LVH, and LVE vs. non-LVE) and those variables were entered into multivariate Cox regression models for the assessment of independent risk factors of LVH or LVE. As shown in Table 3, CBP (per 10mmHg; HR 1.094; 95%CI 1.011–1.202), BMI˃25(kg/m ^2^) (HR 1.336; 95%CI 1.195–1.494), BNP ≥ 100 (pg/mL) (HR 1.635; 95%CI 1.107–3.311) and baPWV ≥16 (m/s) (HR 1.605; 95%CI 1.474–2.039) were associated with the development of LVH. Independent predictors of LVE included CBP (per 10mmHg; HR 1.121; 95%CI 1.027–1.238), BMI˃25(kg/m ^2^) (HR 1.302; 95%CI 1.173–1.444), LDL-C (HR 1.193; 95%CI 1.013–1.405) and E/e’ ratio (HR, 1.077; 95%CI 1.017–1.140), as demonstrated in Table 4.

**Table 3:** Multivariate model for prediction of LVH

Variables	Hazard	Ratio	95%
Age (years)	1.032	0.912–1.108	0.248
Hypertension (%)	1.233	0.610–2.491	0.560
CBP (per 10mmHg)	1.094	1.011–1.202	0.021
BMI ≥25 (kg/m^2^)^a^	1.306	1.175–1.434	<0.001
BNP ≥100 (pg/mL)^b^	1.635	1.107–3.311	0.007
hsCRP (mg/L)	1.009	0.771–1.123	0.664
baPWV ≥16 (m/s)	1.605	1.474–2.039	0.009

Values expressed as Hazard Ratio and 95% confidence interval (CI). Abbreviations are the same as in Table 1

Covariates in the basic model included age, hypertension, CBP, BMI ≥25 (kg/m^2^), BNP ≥ 100 (pg/mL), hsCRP and baPWV ≥16 (m/s)

^a^BMI greater than 25 is considered overweight and formed into two categories according to NICE Guidance 2014

^b^BNP was formed into two categories according to European Society of Cardiology Guidelines for the diagnosis and treatment of acute and chronic heart failure 2012

**Table 4:** Multivariate model for prediction of LVE

Variables	Hazard	Ratio	95%
Age (years)	1.012	0.962–1.066	0.338
Hypertension (%)	1.282	0.725–2.888	0.550
CBP (per 10mmHg)	1.121	1.027–1.238	0.004
BMI ≥25 (kg/m^2^)	1.302	1.173–1.444	<0.001
LDL-C	1.193	1.013–1.405	0.034
hsCRP (mg/L)	1.068	0.967–1.180	0.195
baPWV ≥16 (m/s)	1.573	0.869–2.568	0.159
E/e’ ratio	1.077	1.017–1.140	0.011

Values expressed as Hazard Ratio and 95% confidence interval (CI). Abbreviations are the same as in Tables 1 and 2

Covariates in the basic model included age, hypertension, CBP, BMI ≥25 (kg/m^2^), LDL-C, hsCRP, baPWV ≥16 (m/s) and E/e’ ratio

## Discussion

In this longitudinal cohort study among elderly women, there was a high incidence of new-onset LVE (11.6%) and LVH (10.3%) occurrence. The increase in the levels of CBP and BMI were predictive for both LVE and LVH, after accounting for various potential confounding factors. BaPWV index using detailed noninvasive evaluation and BNP were specifically correlated with the development of LVH, but not LVE. In addition, the E/e’ ratio using Doppler echocardiogram and LDL-C were predictive for the development of LVE.

The effect of gender during cardiac remodeling is still unclear. Data from Framingham Heart Study revealed that women have a greater age-related increase in LV wall thickness [13]. It has also been reported that concentric remodeling is more pronounced in women [14, 15]. To our knowledge, our study is the first which examines the predictors of LV remodeling among elderly women. We had previously reported that among women (age > 40 years), the prevalence of LVH and LVE were 9.34% and 10.04%, respectively [8]. Our current findings show a relatively high incidence rate for both LVH and LVE in elderly women. This could be partly due to the changes in hormone levels in post-menopause women. The cardio-protective role of estrogen has been well established. However, the high prevalence of LVH and LVE might not be entirely due to the depletion of estrogen, but also the expression of testosterone, which promotes cardiac dysfunction and remodeling [16]. Studies have demonstrated that women are less likely to receive preventive treatment or guidance, such as lipid-lowering drugs, aspirin, and therapeutic lifestyle changes [17]. Thus, inadequate control of risk factors in women is a potential risk factor for the occurrence of LV remodeling.

Increased BMI and BP are commonly recognized as predictors of cardiovascular disease. Our study further strengthened the notion that BMI and CBP were the important predictors of LV remodeling, and were strongly related to the development of both LVH and LVE. These findings suggest that effective control of body weight and BP are crucial in the early prevention of LV remodeling. We had previously reported that BNP was a risk factor for both LVH and LVE in the general population [8]. However, our current regression model showed that BNP failed to independently predict for LVE. BNP is elevated in response to an increase in myocardial wall stress. Early mild ventricular dilation plays a protective role and helps maintain an adequate stroke volume [18]. Therefore, this cardiac compensatory function would not lead to significant increases in BNP level following development of LVE. Due to the difference in study population, this result may be explained in part by the gender-specific control of myocardial adaptations to hemodynamic overload. To support this notion, studies have revealed a rapid induction of plasma BNP and ANP during myocardial dysfunction in men [19].

Dyslipidemia is the metabolic abnormalities observed frequently in patients with heart diseases. Francesco et al reported that unsatisfactory control of LDL cholesterol after myocardial infarction independently predicts LV remodeling [20]. For the current study, we found that high LDL-C predicted the development of left ventricular enlargement, but not hypertrophy in multivariable models. Due to the limited data on the relationship between LDL-C and left ventricular remodeling, it is still different to tell whether high LDL-C specifically predicts LVE. This result is broadly consistent with the findings of previous researches [20, 21]. Also, in some sense, it strengthens the need for the achievement and maintenance of target serum lipids for reducing LV remodeling.

The ratio of early trans-mitral flow velocity (E) to early diastolic septal mitral annulus velocity (E/e’) has been shown to be the most accurate noninvasive predictor of elevated LV filling pressure [22]. According to the echocardiography-Doppler criteria for assessment of diastolic function, E/e’>10 indicate moderate to severe diastolic dysfunction [23]. During pressure overload, hearts of female subjects often develop concentric hypertrophy as a compensatory function in order to maintain stable cardiac function [18]. However, when the impairment progresses past a certain stage, ventricular dilation takes place. Our results demonstrated that E/e’ was a predictor of LVE in elderly female subjects. These patients often do not report adverse symptoms, however they are at risk to more serious deterioration of cardiac function. Whether E/e’ and LVIDd is directly proportional is still unknown, and requires further investigation.

Cardiac abnormalities in adults often develop in concert with atherosclerosis [24]. BaPWV is a promising technique to assess arterial stiffness in large population-based investigations [25]. Numerous studies have reported a positive correlation between increased LVMI and baPWV [26, 27]. Arterial stiffness may contribute to LVH independently of BP [26, 28]. Our current study showed that risk of LVH rose markedly in patients with baPWV>16m/s. Increased vascular resistance with hypertension places severe strain on the heart, which is by far the most common cause of LVH [29]. High baPWV level is a common sign of atherosclerotic vascular damage. Thus, evaluation of baPWV is particularly important in elderly women, as a convenient and early indicator of LVH.

This study has several limitations that need to be addressed. Firstly, the prevalence of LVH and LVE might be overstated since the use of echocardiography as a routine screening strategy for cardiac remodeling in population-based investigation remains controversial. LV mass calculated according to the measurements of echocardiography may be overestimated when compared with the gold standard of MRI [30]. Echocardiography is now listed as a class I evidence for the evaluation of patients with HF, while also being widely used for observing alterations in LV geometry and structure [7, 9]. Considering the convenience and practicability of echocardiography in population-based epidemiological investigations, it is the preferred choice for our research design. Secondly, in order to avoid potential bias of information obtained from the self-administered questionnaire, we did not analyze effects of drug use and postpartum hemodynamic changes which may potentially influence LV geometry. The data presented herein were grouped as the presence or absence of LVE (and LVH). Eighteen subjects were confirmed with both LVE and LVH according to the assessment criteria. We fell short of a combinatorial assessment these two remodeling patterns. This needs to be compensated in our future investigations.

## Conclusion

This study shows the incidence rates of new onset LV remodeling in a community-based nested case-control cohort. Risk profiles at baseline for LVH and LVE were determined. We determined that increased LV remodeling may be associated with higher incidence of HF with preserved ejection fraction (HFpEF) in elderly women. Therefore, better understanding of LV remodeling in women is critical in improving the prevention of HF.

## Abbreviations

A: Peak flow velocity of atrial contraction
baPWV: Brachial-ankle pulse wave velocity
BNP: B-type natriuretic peptide
CAD: Coronary artery disease
CBP: Central blood pressure
E: Peak early diastolic flow velocity
e’: Velocity of mitral annulus early diastolic motion
FPG: Fasting plasma glucose
HbA1C: Glaciated hemoglobin
HCY: Homocysteine
HDL-C: High-density lipoprotein cholesterol
HF: Heart failure
hsCRP: High-sensitivity C-reactive protein
IVST: Interventricular sepal thickness
LAD: Left atrium diameter
LDL-C: Low-density lipoprotein cholesterol
LVE: Left ventricular enlargement
LVEF: Left ventricular ejection fraction
LVH: Left ventricular hypertrophy
LVIDd: Left ventricular internal diameter at end-diastole
LVM: Left ventricular mass
LVMI: Left ventricular mass index
PVWT: Posterior left ventricular wall thickness
TC: Total cholesterol
TG: Total triglyceride
UA: Serum uric acid
VHD: Valvular heart disease
